# ASPM promotes hepatocellular carcinoma progression by activating Wnt/β‐catenin signaling through antagonizing autophagy‐mediated Dvl2 degradation

**DOI:** 10.1002/2211-5463.13278

**Published:** 2021-09-14

**Authors:** Haifeng Zhang, Xiaobei Yang, Lili Zhu, Zhihui Li, Peipei Zuo, Peng Wang, Jingyu Feng, Yang Mi, Chengjuan Zhang, Yan Xu, Ge Jin, Jianying Zhang, Hua Ye

**Affiliations:** ^1^ Department of Biochemistry & Molecular Biology School of Basic Medical Sciences Zhengzhou University China; ^2^ Academy of Medical Sciences Zhengzhou University China; ^3^ Henan Institute of Medical and Pharmaceutical Sciences Zhengzhou University China; ^4^ Center of Repository The Affiliated Cancer Hospital of Zhengzhou University China; ^5^ College of Public Health Zhengzhou University China

**Keywords:** ASPM, autophagy, Dvl2, hepatocellular carcinoma, Wnt/β‐catenin

## Abstract

Hepatocellular carcinoma (HCC) is one of the most fatal cancers worldwide. In this article, we show that expression of abnormal spindle‐like microcephaly‐associated protein (ASPM) is up‐regulated in liver cancer samples, and this up‐regulation is significantly associated with tumor aggressiveness and reduced survival times of patients. Down‐regulation of ASPM expression inhibits the proliferation, invasion, migration and epithelial‐to‐mesenchymal transition of HCC cells *in vitro* and inhibits tumor formation in nude mice. ASPM interacts with disheveled‐2 (Dvl2) and antagonizes autophagy‐mediated Dvl2 degradation by weakening the functional interaction between Dvl2 and the lipidated form of microtubule‐associated proteins 1A/1B light chain 3A (LC3II), thereby increasing Dvl2 protein abundance and leading to Wnt/β‐catenin signaling activation in HCC cells. Thus, our results define ASPM as a novel oncoprotein in HCC and indicate that disruption of the Wnt–ASPM–Dvl2–β‐catenin signaling axis might have potential clinical value.

## Graphical abstract

The abnormal spindle‐like microcephaly‐associated protein (ASPM) promotes hepatocellular carcinoma progression by activating Wnt/β‐catenin signaling. ASPM interacts with disheveled‐2 (Dvl2) and antagonizes autophagy‐mediated Dvl2 degradation by weakening the functional interaction between Dvl2 and the lipidated form of microtubule‐associated proteins 1A/1B light chain 3A (LC3II), thereby increasing Dvl2 protein abundance and leading to Wnt/β‐catenin signaling activation in HCC cells.

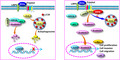

**Abbreviations**
ASPM: abnormal spindle‐like microcephaly‐associated
CCK‐8: Cell Counting Kit‐8
Co‐IP: coimmunoprecipitation
DMEM: Dulbecco's modified Eagle's medium
Dvl2: disheveled‐2
ECL: enhanced chemiluminescence
EMT: epithelial‐to‐mesenchymal transition
HBV: hepatitis B virus
HBV‐HCC: hepatitis C virus‐related primary hepatocellular carcinoma
HBV‐RHCC: hepatitis C virus‐related recurrent hepatocellular carcinoma
HCC: hepatocellular carcinoma
KD: knockdown
LC3II: lipidated form of microtubule‐associated proteins 1A/1B light chain 3A
LEF1: lymphoid enhancer factor 1
MLC: metastatic liver cancer
shCtrl: scrambled control
shRNA: short hairpin RNA
shASPM: shRNA against ASPM
TCF4: T cell factor 4

Hepatocellular carcinoma (HCC) is the fifth most common tumor worldwide and the second leading cause of cancer‐related mortality [1]. The incidence of HCC has been increasing rapidly over the last 20 years [1]. The current available therapies for patients with advanced HCC are extremely limited, hence resulting in a very low 5‐year survival rate [2]. Even with the advent of the molecular targeted agents, sorafenib and regorafenib, prolonged survival is limited [3, 4]. Thus, it is imperative to deeply explore the underlying molecular mechanisms of HCC pathogenesis and identify novel targets and/or prognosis predictors to improve the therapeutic outcome.

The abnormal spindle‐like microcephaly‐associated protein (ASPM) consists of 3477 amino acids and distributes in the spindle poles during mitosis and centrosome during interphase [5]. ASPM exerts critical roles in the symmetric divisions of neural progenitor cells of the mammalian brain [6], and mutations within *ASPM* gene are the most common cause of familial microcephaly [7, 8]. ASPM forms a complex with the microtubule severing ATPase katanin to control microtubule minus‐end disassembly at spindle poles [9]. ASPM also acts as a cell fate‐determining factor by regulating cyclin E abundance [10]. However, recent studies have shown that ASPM may promote cell proliferation and be involved in various human cancers [11, 12].

ASPM expression is incrementally up‐regulated in prostate cancer, and the increased expression of ASPM correlates with tumor progression and poor clinical prognosis [13, 14]. The depletion of ASPM by siRNA results in dramatic proliferation arrest of neural stem cells and glioblastoma cells, supporting ASPM as a potential molecular target in the treatment of glioblastoma [15, 16]. ASPM enhances the repair of DNA double‐strand breaks, and the combination of ASPM‐specific siRNA with radiotherapy promotes the radiosensitivity in immortalized human cell lines [17]. In pancreatic ductal adenocarcinoma, ASPM is negatively associated with glandular differentiation and promotes stem cell features and tumorigenic potential [18]. Recent study showed that ASPM isoforms 1 and 2 have different expression patterns and functions in pancreatic cancer [19]. Moreover, ASPM is overexpressed in human gastric adenocarcinomas and may represent a potential marker of gastric progenitor cells [20].

Bioinformatics analysis and RT‐PCR results show that the expression of ASPM is increased in HCC and is associated with poor prognosis in patients with HCC [21, 22], but its biological function and molecular mechanisms in HCC are still unknown. In this study, we report that ASPM expression is up‐regulated, at both mRNA and protein levels, in liver tumor tissues, and up‐regulation of ASPM is related to aggressive clinicopathological features and patient's short overall survival. The short hairpin RNA (shRNA)‐mediated knockdown (KD) of ASPM inhibits HCC cell proliferation, migration, invasion and epithelial‐to‐mesenchymal transition (EMT) *in vitro* and attenuates the tumorigenesis of HCC *in vivo*. Mechanistic investigations showed that ASPM augments Wnt–disheveled‐2 (Dvl2)–β‐catenin signaling by directly binding to and stabilizing Dvl2 via antagonizing the autophagy system in HCC cells.

## Materials and methods

### Patients and tissue samples

Specimens including 102 liver cancer and nontumor adjacent tissues were collected from patients who received hepatectomy of liver cancer in the Affiliated Cancer Hospital of Zhengzhou University (Zhengzhou, China) from July 2016 to December 2018, of which 90 samples were paired. The specimens included 74 hepatitis B virus (HBV)‐related primary HCCs (HBV‐HCCs), 10 HBV‐related recurrent HCCs (HBV‐RHCCs), 4 primary HCCs, 7 intrahepatic cholangiocarcinomas and 7 metastatic liver cancers (MLCs). The detailed information of samples is shown in Table S1. The protocol was approved by the ethics committee of Zhengzhou University, and written informed consent was obtained from each patient. The study methodologies conformed to the standards set by the Declaration of Helsinki.

### Cell culture

The human cell lines HEK293T, LO2, SMMC7721, HepG2, MHCC97H and Huh1 were kind gifts from P. Xu (State Key Laboratory of Proteomics, Beijing Proteome Research Center, National Center for Protein Sciences, Beijing, China) and T. Liu (Sino‐American Hormelian Cancer Research, Zhengzhou, China), and human HCC cell line LM3 was purchased from China Center for Type Culture Collection (Wuhan, China). All cell lines were routinely cultured at 37 °C with 5% CO_2_ in Dulbecco's modified Eagle's medium (DMEM; BI, Shanghai, China) supplemented with 10% FBS, 100 μg·mL^−1^ streptomycin and 100 U·mL^−1^ penicillin.

### Generation of stable KD cells and plasmid transient transfection

Lentiviral vector encoding shRNA against ASPM (shASPM) and scrambled control (shCtrl) were purchased from GeneCopoeia Company (Guangzhou, China). shASPM or shCtrl vectors along with packaging plasmids (Lenti‐Pac™ HIV; GeneCopoeia Company) were cotransfected into HEK293T cells using Lipofectamine 2000. Lentiviral particles were collected and concentrated at 48 h after transfection. HepG2 and SMMC7721 cells were infected with the virus in the presence of 8 μg·mL^−1^ polybrene, and the infected cells were selected by puromycin at 2 μg·mL^−1^. To overexpress Dvl‐2, we transiently infected the full‐length Dvl‐2 plasmid (GV417‐Dvl‐2) into shASPM HCC cells using Lipofectamine 2000. GV417 was used as a negative control (GeneChem, Shanghai, China).

### Cell Counting Kit‐8 assay

For the proliferation assay, 5 × 10^3^ cells per well were seeded in 96‐well plates. At the appointed time, 10 μL Cell Counting Kit‐8 (CCK‐8) reagent (Dojindo Laboratories, Kumamoto, Japan) was supplemented into each well, and the absorbance was calculated for each well at 450 nm (*A*_450 nm_) with a microplate spectrophotometer (BioTek, Winooski, VT, USA).

### Cell migration assay

Cell migration was examined using a scratch wound healing assay. HCC cells were grown to confluence in six‐well plates, and a scratch line was made to the cell monolayer using a sterile 10‐μL pipette tip. The cells were washed twice with PBS to eliminate floating cells and exchanged with serum‐free DMEM. Wound images were taken, and the width of the wound gaps was measured using Image J Software (National Institutes of Health, Bethesda, MA, USA).

### Cell invasion assay

Cell invasion capacity was measured by BioCoat Matrigel invasion chambers (Corning Inc., Corning, NY, USA). In brief, 100 μL Matrigel was supplemented into each transwell chamber. A total of 5 × 10^4^ cells suspended in serum‐free medium were added to the upper chambers, and 600 μL DMEM containing 10% FBS was added in the lower chamber. After incubation at 37 °C for 36 h, Matrigel attached to the membranes was carefully removed. The membranes were fixed with 4% paraformaldehyde and stained with 4′,6‐diamidino‐2‐phenylindole (DAPI). The cells were photographed by fluorescence microscopy and counted in five random fields of microscope.

### Tumor xenografts

BALB/c male nude mice 4 weeks of age were purchased from the Beijing Vital River Laboratory Animal Technology Company. Five mice per cage were housed in individually ventilated cage systems with standard dark/light intervals (12 h/12 h), and all mice had *ad libitum* access to autoclaved food and water. The animal room was maintained at 26 °C with a humidity of 60%, and the bedding was changed weekly with autoclaved pine shavings. A total of 1 × 10^6^ HepG2‐shCtrl cells or HepG2‐shASPM cells were injected subcutaneously into the right flanks of 4.5‐week‐old mice. Each group contained six mice. Tumor growth was monitored every 3 days. Tumor volume was calculated with the formula of (0.5 × length × width^2^). All animal experiments were performed in accordance with the *Guidelines for the Care and Use of Laboratory Animals* and were approved by the Animal Ethics Committee of Zhengzhou University.

### TOP/FOPFlash reporter assay

After incubation at 37 °C for 24 h, HCC‐shASPM or HCC‐shCtrl cells in 24‐well plates were transfected with TOPFlash or FOPFlash plasmid (800 ng·well^−1^) and pRL‐TK plasmid (50 ng·well^−1^) using Lipofectamine 2000. Luciferase and Renilla activities were measured 48 h after transfection by the Dual Luciferase Reporter Assay Kit (Promega, Madison, WI, USA). The luciferase activity of each sample was normalized with the respective Renilla activity.

### Quantitative RT‐PCR

Total RNA was isolated using an RNAiso Plus kit (Takara Bio Inc, Da Lian, China). The cDNA for quantitative RT‐PCR (qRT‐PCR) was synthesized from 1 μg of total RNA using a PrimeScript RT reagent kit (Takara Bio Inc.). The mRNA expression was detected by two‐step quantitative reverse transcription PCR using an ABI 7500 Fast Real‐Time PCR system. *GAPDH* was used as a reference gene, and the expression of target mRNA was calculated using the 2^−ΔCT^ method. The primers used are listed in Table S2.

### Western blotting

Cells were lysed with radio immunoprecipitation assay lysis buffer supplemented with protease inhibitor cocktail (CW2200s; CWBIO, Beijing, China) and phosphatase inhibitors. After denaturation with SDS/PAGE loading buffer for 5 min at 95 °C, proteins were separated via 10% SDS/PAGE (for ASPM, 6% SDS/PAGE) and then transferred onto poly(vinylidene difluoride) membrane. The membrane was blocked in 5% skim milk powder and incubated with primary antibodies at 4 °C overnight. After washing with TBST, the membrane was incubated with secondary antibodies for 2 h at room temperature. Finally, the membrane was filmed with enhanced chemiluminescence solution (ECL), and the ECL detection system (FluorChem E, ProteinSimple, Santa Clara, California, USA) was used to visualize the signal. Antibodies used included ASPM (H‐141, sc‐98903, 1 : 500 dilution), Dvl‐2 (D‐6, sc‐390303, 1 : 1000 dilution), Dvl1 (3F12, sc‐8025, 1 : 1000 dilution), Dvl3 (4D3, sc‐8027, 1 : 1000 dilution), T cell factor 4 (TCF4) (D‐4, sc‐166699, 1 : 3000 dilution), Axin (2B11, sc‐293190, 1 : 3000 dilution), lymphoid enhancer factor 1 (LEF1) (B‐10, sc‐374412, 1 : 3000 dilution), LC3‐II (G‐2, sc‐271625, 1 : 2000 dilution), P62 (D‐3, sc‐28359, 1 : 3000 dilution), glyceraldehyde‐3 phosphate dehydrogenase(GADPH) (0411, sc‐47724, 1 : 7000 dilution), ‐β‐actin (C4, sc‐47778, 1 : 7000 dilution; all from Santa Cruz Biotechnology, Santa Cruz, CA, USA); β‐catenin (66379‐I‐Ig, 1 : 3000 dilution) and c‐Myc (10828‐I‐AP, 1 : 3000 dilution; Proteintech, Wuhan, China); and Glycogen synthase kinase‐3β (GSK‐3β) (200494‐2E6, 1 : 3000 dilution; Zen Bioscience, Chengdu, China).

### Coimmunoprecipitation experiment

The protein extraction and quantification are the same as western blotting assay. A total of 800 μg of total protein was incubated with protein A/G agarose (SC2003; Santa Cruz) in a ratio of 20 μL for a 1‐ml sample solution at 4 °C for 30 min and then centrifuged at 1006 *g*   for 30 s to remove nonspecific protein. The supernatant was incubated with 2 μg of primary antibodies at 4 °C overnight and then added with 40 μL protein A/G agarose at 4 °C for 4 h. Next, the beads were collected by centrifuging at 1006 *g*  for 30 s and washed with radioimmunoprecipitation assay lysis buffer. The bound proteins were subjected to immunoblotting analysis. The mouse IgG was used as a negative control.

### Immunohistochemistry

The paraffin‐fixed liver tissues were sectioned and dewaxed. After antigen retrieval, tissue sections were incubated with primary antibody against ASPM (H‐141, sc‐98903; 1 : 200 dilution; Santa Cruz) at 4 °C overnight. After washing with PBS, tissue sections were incubated with enhanced HRP‐conjugated secondary antibody polymer (pv‐9000; ZSGB‐Bio, Beijing, China) and subsequently treated with 2,4‐diaminobutyric acid solution and then counterstained with hematoxylin. For the negative control, PBS was used to replace the primary antibody.

### Immunofluorescence cell staining

After 24‐h culture, cells on the coverslips in the 24‐well plates were fixed with 4% paraformaldehyde and permeabilized with 0.5% Triton X‐100. After blocking with 0.5% BSA, the cells were incubated with rabbit polyclonal antibody ASPM (1 : 50 dilution, SC‐98903; Santa Cruz) and mouse monoclonal antibody Dvl‐2 (1 : 50 dilution, sc‐390303; Santa Cruz) at 4 °C overnight and then with fluorescence secondary antibodies (Alexa Fluor 594, SA00006‐3, 1 : 200 dilution; FITC, SA00003‐2, 1 : 200 dilution; Proteintech) at room temperature for 2 h. The nuclei were stained with DAPI for 2 min and washed with PBS. Finally, coverslips were mounted, and images of cells were observed by using the fluorescence microscope (Olympus, Tokyo, Japan).

### Statistical analysis

The normality of the data distribution was assessed using the Kolmogorov–Smirnov and Shapiro–Wilk methods. A two‐tailed Student's *t*‐test was used to compare two groups with normal distribution data. For non‐normal distribution data, the Mann–Whitney *U* test was used for pairwise comparisons. Nonparametric Spearman’s rank correlation analysis was performed to calculate the correlation coefficient (*r*). Kaplan–Meier plots were used for analysis of overall survival data. For the ASPM expression level analyses, the cutoff value for defining the subgroups was the median. A *P* value <0.05 was considered statistically significant (two‐tailed). spss Statistics 17 software (SPSS Inc, Chicago, IL, USA) was used for statistical analyses. Graphs were generated using graphpad prism 5.0 software (GraphPad Software Inc., La Jolla, California).

## Results

### ASPM expression is up‐regulated in liver cancer tissues and correlates with malignant clinicopathologic features and poor prognosis

**Fig. 1:**
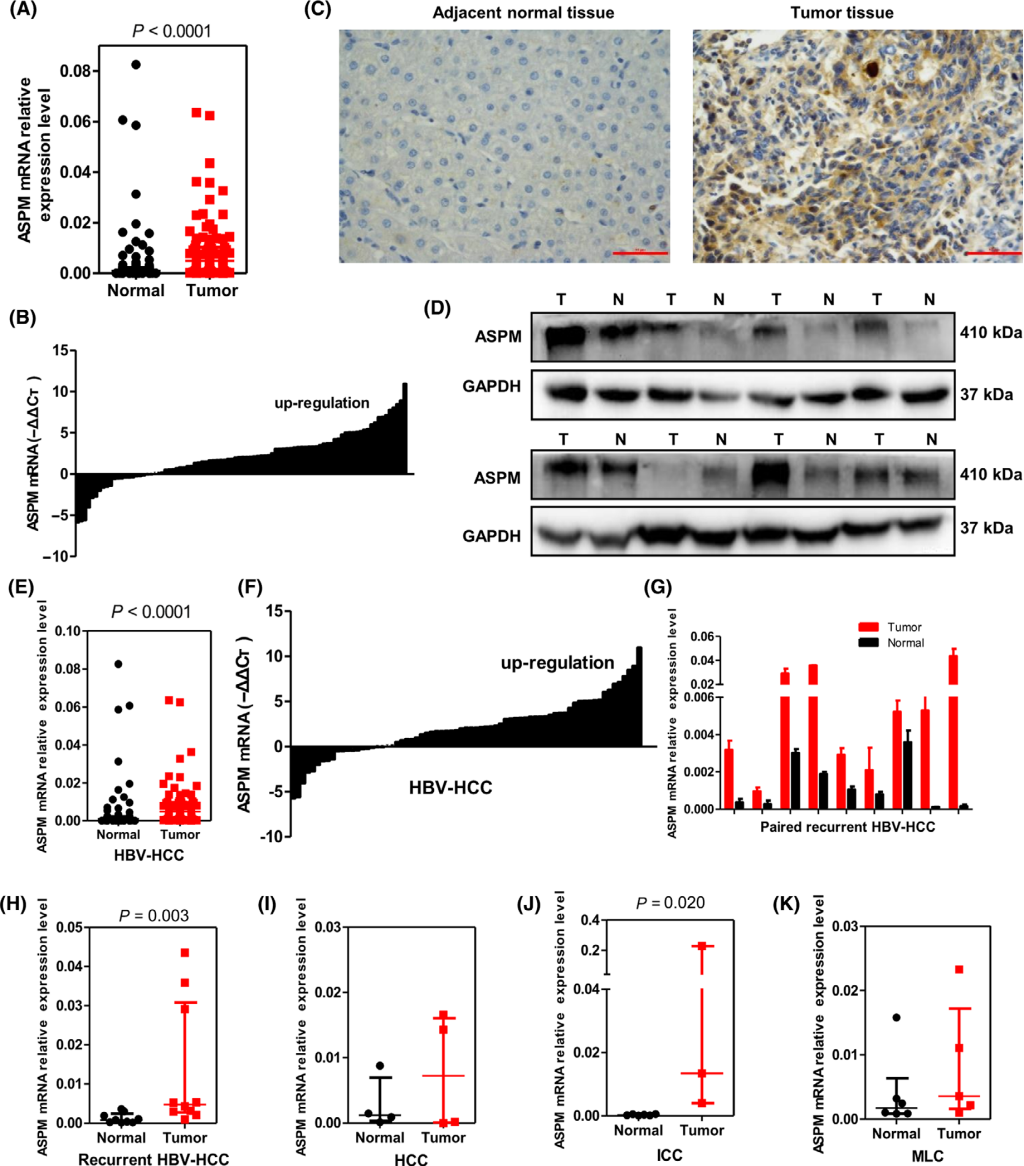
ASPM level is up‐regulated in liver tumor tissues. (A) The expression levels of ASPM mRNA in liver tumor tissues and adjacent nontumor tissues were evaluated by qRT‐PCR (*n* = 102). (B) The expression levels of ASPM mRNA in 90 paired liver tumor tissues and adjacent nontumor tissues were evaluated by qRT‐PCR. (C) Representative images of ASPM immunohistochemistry in paired adjacent nontumor and liver tumor tissues. Scale bars: 50 μm. (D) The protein levels of ASPM in paired liver tumor (T) and adjacent nontumor (N) tissues measured by western blot assay. (E, F) The expression levels of ASPM mRNA in HBV‐HCC tumor tissues and adjacent nontumor tissues (*n* = 74), as well as in 70 paired HBV‐HCC tumor tissues and adjacent nontumor tissues, were evaluated by qRT‐PCR. (G) The expression levels of ASPM mRNA in nine paired HBV‐RHCC tumor tissues and adjacent nontumor tissues were evaluated by qRT‐PCR. Data are mean ± SD, *n* = 3, Student's *t*‐test. (H–K) The expression levels of ASPM mRNA in HBV‐RHCC (H, *n* = 10), HCC (I, *n* = 4), intrahepatic cholangiocarcinoma (ICC) (J, *n* = 6) and MLC (K, *n* = 6) tumor tissues and their respective adjacent nontumor tissues. Data are presented as median with interquartile range, and the Mann–Whitney *U* test was used for pairwise comparisons (A, E, H–K).

ASPM mRNA were determined by qPCR in a total of 102 human liver samples, in which 90 specimens were paired. As shown in Fig. 1A, the expression of ASPM mRNA was significantly up‐regulated in liver cancer tissues compared with that in adjacent nontumor tissues (*P* < 0.0001). In the paired tumor and adjacent nontumor tissues, ASPM mRNA overexpression was found in 69 of 90 (76.7%) samples (Fig. 1B). We next performed western blot and immunohistochemistry staining in 65 and 32 paired samples, respectively. The protein expression of ASPM was higher in 73.8% (48/65) and 71.9% (23/32) of tumor tissues, respectively (Fig. 1C,D).

The 102 human liver samples were obtained from different histological types or different phases of liver cancer patients and were divided into five groups (Table S1). The median levels of ASPM mRNA expression in tumor tissues of all five groups were higher than those in the adjacent nontumor tissues, although they were not statistically significant in HCC and MLC groups because of limited samples (Fig. 1E–K and Table S3). Moreover, comparing with that in adjacent nontumor tissues, ASPM mRNA levels in all of the nine matched HBV‐RHCC tumor samples were increased (Fig. 1G; *P* = 0.003), implicating its potential role in the recurrence of HBV‐HCC.

**Fig. 2:**
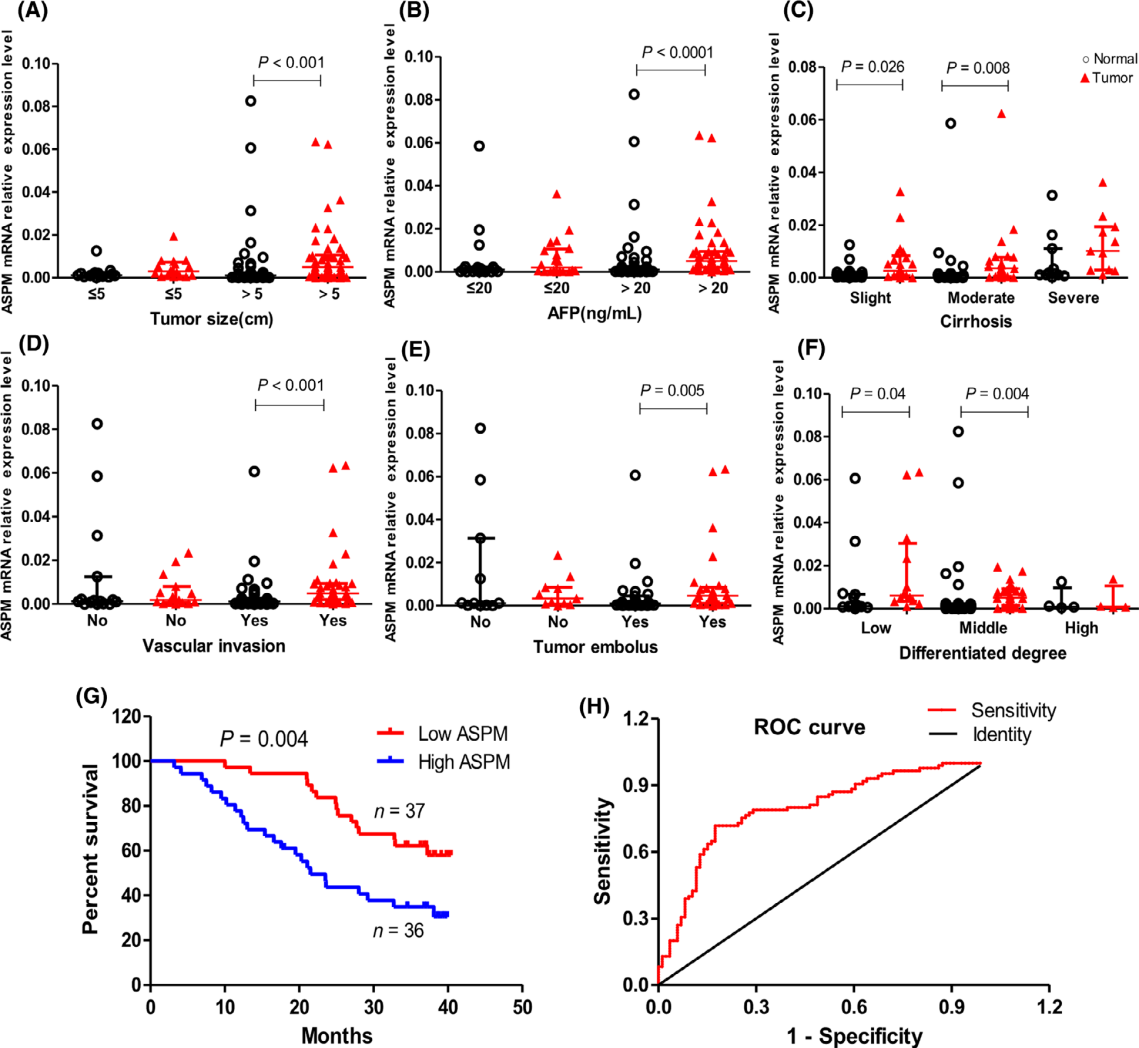
Up‐regulation of ASPM is positively associated with malignant clinicopathologic features of patients with liver cancer. (A–F) The expression levels of ASPM mRNA in liver tumor tissues and adjacent nontumor tissues were evaluated by qRT‐PCR according to tumor size (A: ≤5 cm, *n* = 16; >5 cm, *n* = 54), AFP level (B: ≤20 ng·mL^−1^ *n* = 20; >20 ng·mL^−1^, *n* = 50), cirrhosis severity (C: slight, *n* = 22; moderate, *n* = 23; severe, *n* = 11), vascular invasion (D: no, *n* = 16; yes, *n* = 36), tumor embolus (E: no, *n* = 11; yes, *n* = 27) and differentiation degree (F: low, *n* = 12; middle, *n* = 30; high, *n* = 4). Data are presented as median with interquartile range, and the Mann–Whitney *U* test is used for pairwise comparisons. (G) Kaplan–Meier analysis of overall survival in patients with liver cancer with variable expression of ASPM. (H) Receiver operating characteristic curve based on ASPM mRNA expression levels, *n* = 90.

The most common risk factor for HCC is viral infection, and 80% of HCC cases are attributable to HBV infection in China [23]. There are 74 primary HBV‐HCCs in our samples, and we next investigated the clinical significance of ASPM in patients with HBV‐HCC. Comparing with the adjacent nontumor tissues, ASPM mRNA levels were significantly higher in HBV‐HCC tumor tissues with large tumor size (>5 cm; *P* < 0.001), high alphafetoprotein (AFP) level (≥20 ng·mL^−1^, *P* < 0.00001), slight (*P* = 0.026) and middle cirrhosis (*P* = 0.008), vascular invasion (*P* < 0.001), tumor embolus (*P* = 0.004), and low (*P* = 0.022) and middle differentiation (*P* = 0.004; Fig. 2A–F). Whereas in groups with small tumor size (≤5 cm), low AFP value (≤ 20 ng·mL^−1^), severe cirrhosis, no vascular invasion, no tumor embolus and high differentiation, there was no difference between tumor tissues and the corresponding nontumor tissues. The earlier data clearly demonstrate that ASPM may promote HBV‐HCC malignant progression.

Kaplan–Meier analysis revealed that the expression of ASPM was significantly associated with overall survival of patients with liver cancer (*P* = 0.004), showing a median survival of >34.0 months in ASPM low‐expression group versus 24.4 months in the ASPM high‐expression group (Fig. 2G). The diagnostic value of ASPM was evaluated using a receiver operating characteristic curve, and the area under the curve reached 0.79 (95% confidence interval: 0.722–0.859; *P* = 0.000; Fig. 2H), indicating that ASPM is a potentially valuable biomarker for diagnosing liver cancer.

### ASPM promotes HCC cell proliferation, invasion and migration both *in vitro* and *in vivo*

**Fig. 3:**
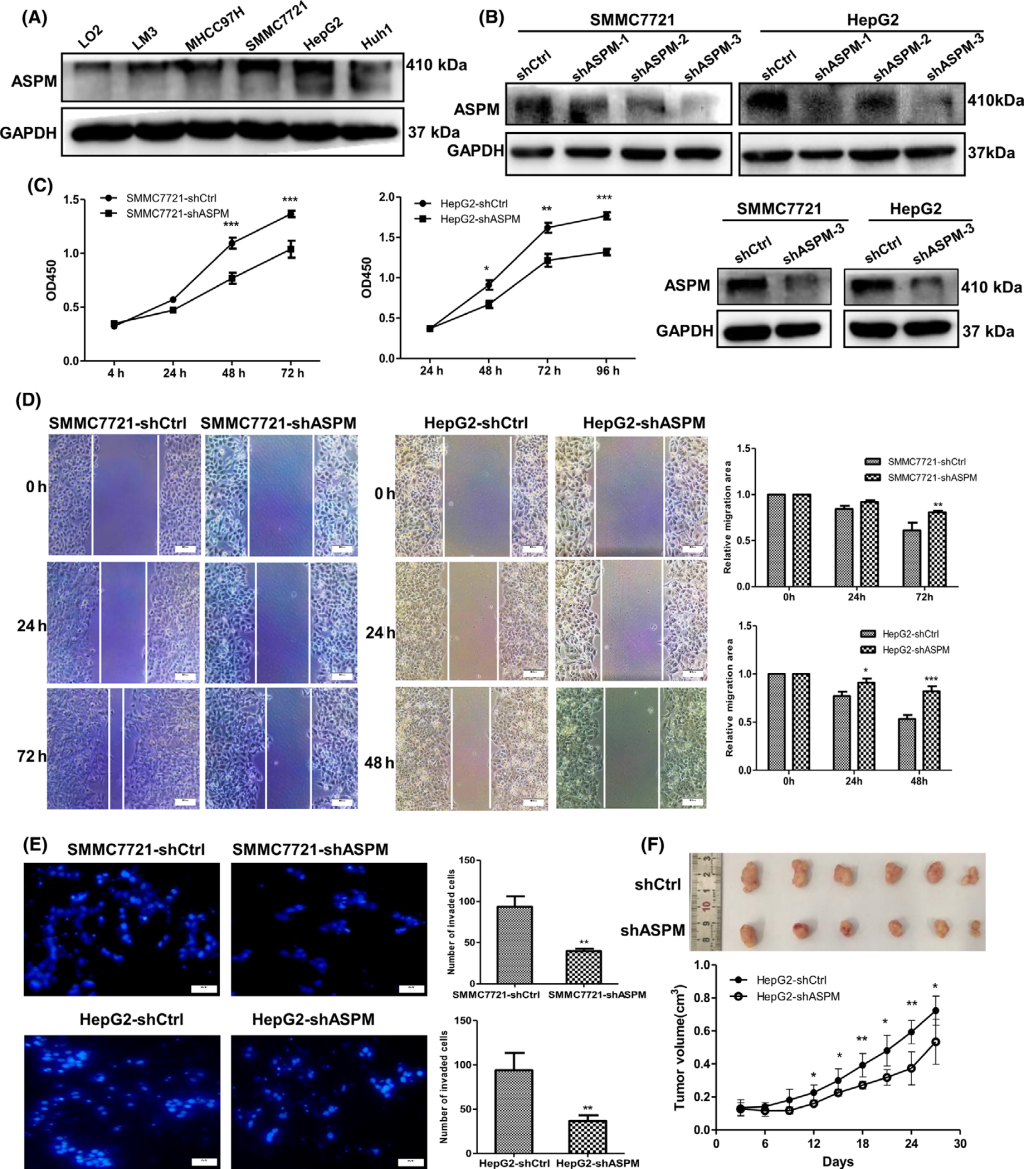
ASPM contributes to the malignant properties of HCC cells. (A) Western blot analysis of ASPM expression in LO2 and various HCC cells. (B) Expression level of ASPM was detected by western blot in ASPM‐KD HCC cells. (C) Proliferation curve of ASPM‐KD SMMC7721, HepG2 and the respective control cells performed by CCK‐8 assay. Data are shown as means ± SD (*n* = 3 in each assay, Student's *t*‐test). *P ≤ 0.05, **P ≤ 0.01, ***P ≤ 0.001 vs. control shRNA. (D) Representative images of scratch wound assays of ASPM‐KD SMMC7721 (left panel), HepG2 (middle panel) and the respective control cells. Scale bars: 100 μm. Data are shown as means ± SD (*n* = 3 in each assay, Student's *t*‐test). *P ≤ 0.05, **P ≤ 0.01, ***P ≤ 0.001 vs. control shRNA (right panel). (E) Representative images of transwell assays of ASPM‐KD SMMC7721, HepG2 and the respective control cells. Scale bars: 100 μm. The results were plotted as the average number of invasive cells from six random microscopic fields. **P ≤ 0.01 vs. control shRNA (right panel). (F) Image and growth curve of xenografts in nude mice injected with HepG2‐shCtrl or HepG2‐shASPM cells. Data are shown as means ± SD (*n* = 6, Student's *t*‐test). *P ≤ 0.05; **P ≤ 0.01; ***P ≤ 0.001 vs. control shRNA.

Given the significant correlation between ASPM expression level and malignant clinicopathologic features in liver cancer samples, we hypothesize that ASPM may play a functional role in the tumorigenesis and progression of liver cancer. We examined a panel of human liver cell lines and found that ASPM expression was higher in HCC‐derived cell lines than in normal liver cell line (L02), which is consistent with the findings in liver tumor tissue samples (Fig. 3A). To assess the functional role of ASPM in HCC cells, we stably knocked down its expression by lentivirus‐mediated shRNA interference in SMMC7721 and HepG2 cells, which have high levels of endogenous ASPM (Fig. 3A). Among three ASPM shRNAs tested, shASPM‐3 resulted in the most significant KD effect in both HCC cells, and it was used for subsequent experiments (Fig. 3B). The CCK‐8 assays showed that the growth rate of shASPM HCC cells was apparently lower than that of the control cells (Fig. 3C). The scratch wounds in shASPM HCC cells changed slightly, while those in control cells narrowed markedly (Fig. 3D). The transwell assays showed that ASPM KD by shRNA significantly reduced the invasion of HCC cells (Fig. 3E).

To verify the consequences of ASPM depletion *in vivo*, we subsequently injected HepG2‐shASPM and HepG2‐shCtrl cells into the right dorsal flank of the nude mice. The results showed that tumors that developed from ASPM‐KD cells were significantly smaller and lighter (Fig. 3F) 27 days after injection. Taken together, the results suggest that ASPM significantly promotes the proliferation, migration and invasion of HCC cells *in vitro* and tumor formation in nude mice.

### ASPM induces EMT of HCC cells

**Fig. 4:**
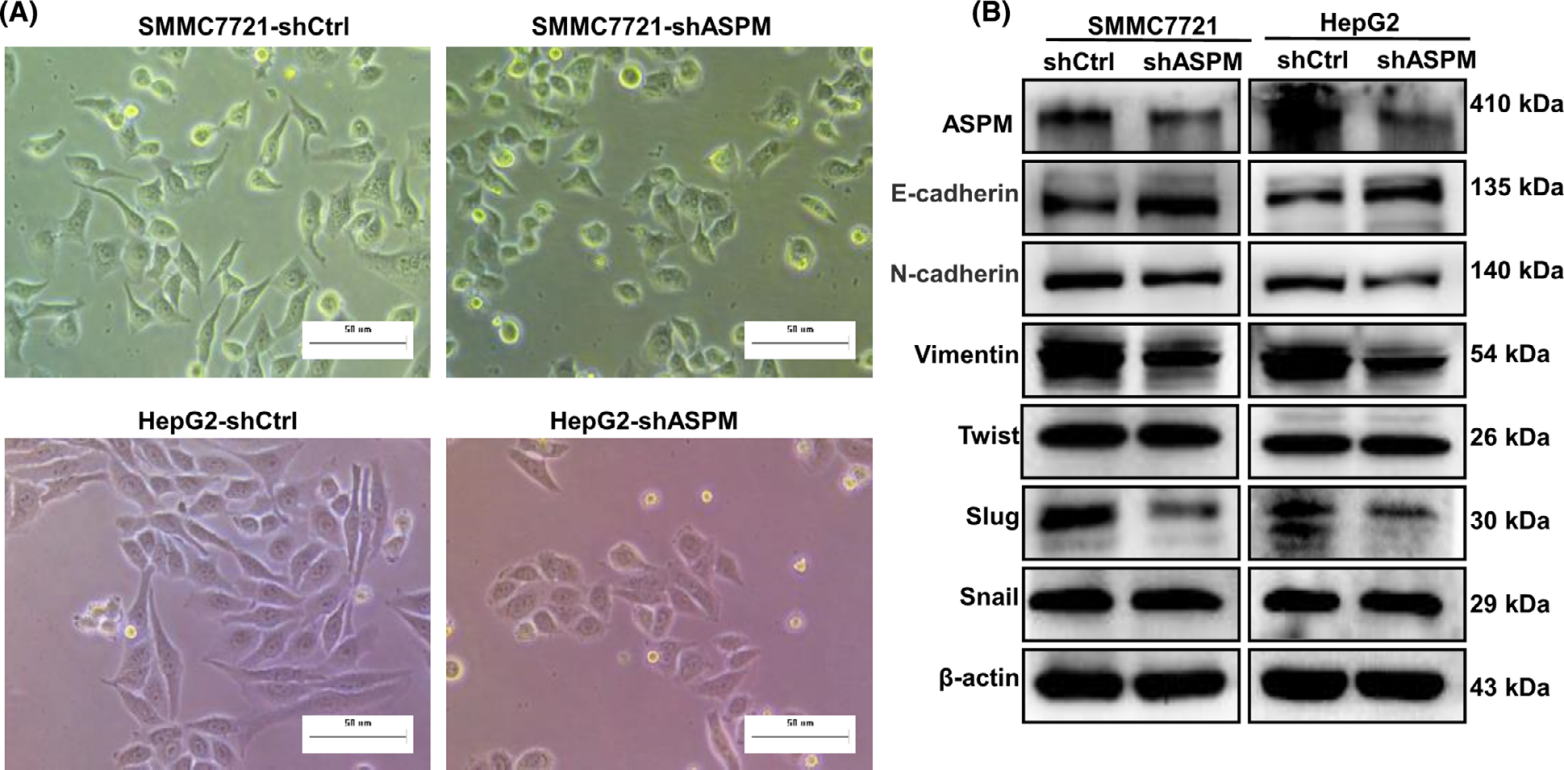
ASPM induces EMT in HCC cells. (A) Representative images of the morphological changes of SMMC7721 and HepG2 after silencing of ASPM by shRNA. Scale bars: 50 μm. (B) Western blot analysis of the protein levels of epithelial and mesenchymal markers (E‐cadherin, Vimentin and N‐cadherin), as well as EMT‐related transcriptional factors (Slug, Twist and Snail) in shCtrl or shASPM SMMC7721 and HepG2 cells.

In this study, we observed an interesting phenomenon in which shASPM HCC cells exhibited cobblestone‐like epithelial morphology (Fig. 4A). Considering the morphologic change in these cells and the function of ASPM, we speculated that ASPM could induce EMT of HCC cells. Western blot results showed that KD of ASPM increased the expression of the epithelial marker E‐cadherin and reduced the expression of the mesenchymal marker N‐cadherin and Vimentin both in HepG2 and SMMC7721 cells (Fig. 4B). Meanwhile, the protein level of EMT‐related transcriptional factors Slug was significantly down‐regulated in shASPM HCC cells, whereas Twist and Snail were almost unchanged (Fig. 4B). Thus, ASPM may induce EMT by modulating Slug expression.

### ASPM activates the Wnt/β‐catenin signaling pathway via interacting with Dvl2 in HCC cells

**Fig. 5:**
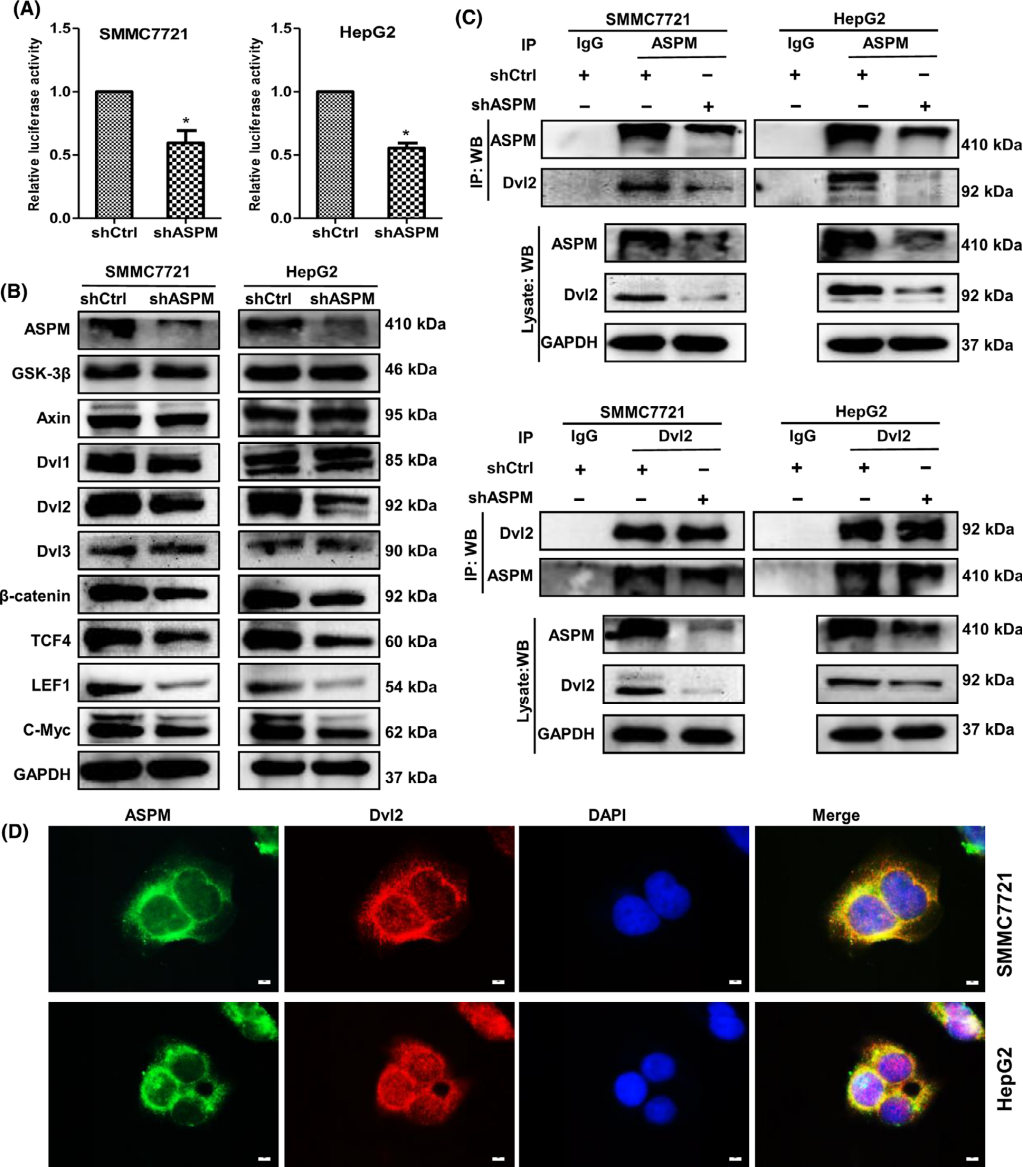
ASPM activates Wnt/β‐catenin signaling by interacting with Dvl2 in HCC cells. (A) Luciferase activity of TOP/FOPFlash in control or ASPM‐KD SMMC7721 and HepG2 cells. Data are shown as means ± SD (*n* = 3 in each assay, Student's *t*‐test). *P ≤ 0.05 vs. control shRNA. (B) Western blot analysis of the protein expression of Wnt/β‐catenin signaling‐related genes, including GSK‐3β, Axin, Dvl‐2, β‐catenin, TCF4, LEF1 and c‐Myc in control or ASPM‐KD SMMC7721 and HepG2 cells. (C) Reciprocal Co‐IP analysis between ASPM and Dvl2 in control or ASPM‐KD SMMC7721 (top panel) and HepG2 cells (bottom panel). (D) Immunofluorescence analysis of colocalization of ASPM (green) and Dvl‐2 (red) in SMMC7721 and HepG2 cells. Scale bars: 10 μm. IP, immunoprecipitation; WB, western blotting.

The Wnt/β‐catenin signaling pathway is frequently activated in HCC [24], and ASPM has recently been revealed as a positive regulator of the Wnt/β‐catenin signaling pathway [13, 25]. We thus hypothesized that ASPM may regulate the activity of the Wnt signaling pathway to facilitate the malignant behaviors of HCC cells. To verify our hypothesis, we first conducted a TOP/FOPFlash reporter assay in ASPM KD and control HCC cells and found that deletion of ASPM significantly inhibited the transcription activity of β‐catenin/TCF4 in both HepG2 and SMMC7721 cells (Fig. 5A). Then, we used western blot to examine the expression of the important Wnt/β‐catenin pathway components in HCC cells. The results demonstrated that ASPM KD markedly decreased the protein levels of disheveled 2 (Dvl2), β‐catenin, TCF4, LEF1 and c‐Myc, and that the upstream regulators of the Wnt pathway, GSK‐3β and Axin, were almost unchanged (Fig. 5B). Interestingly, ASPM KD had no significant effects on the protein levels of Dvl1 and Dvl3 (the other two Dvl isoforms) in both HepG2 and SMMC7721 cells. Dvl2 is the hub of the Wnt signaling pathway, which relays Wnt signals from receptors to β‐catenin by inhibiting its degradation [26, 27]. The earlier western blot results raised the possibility that ASPM may exert its Wnt‐regulatory function through Dvl2. We then investigated whether ASPM and Dvl2 could physically interact by performing coimmunoprecipitation (Co‐IP) assays in both HepG2 and SMMC‐7721 cells. The results showed that ASPM interacted with Dvl2 in reciprocal Co‐IP experiments, and ASPM depletion obviously weakened its interaction with Dvl2 (Fig. 5C). Consistently, colocalization of endogenous ASPM and Dvl2 in the cytosolic compartment near the nuclear membrane was observed in both HepG2 and SMMC‐7721 cells via immunofluorescence microscopy (Fig. 5D). The earlier results demonstrate that ASPM may target Dvl2 to regulate Wnt signaling.

**Fig. 6:**
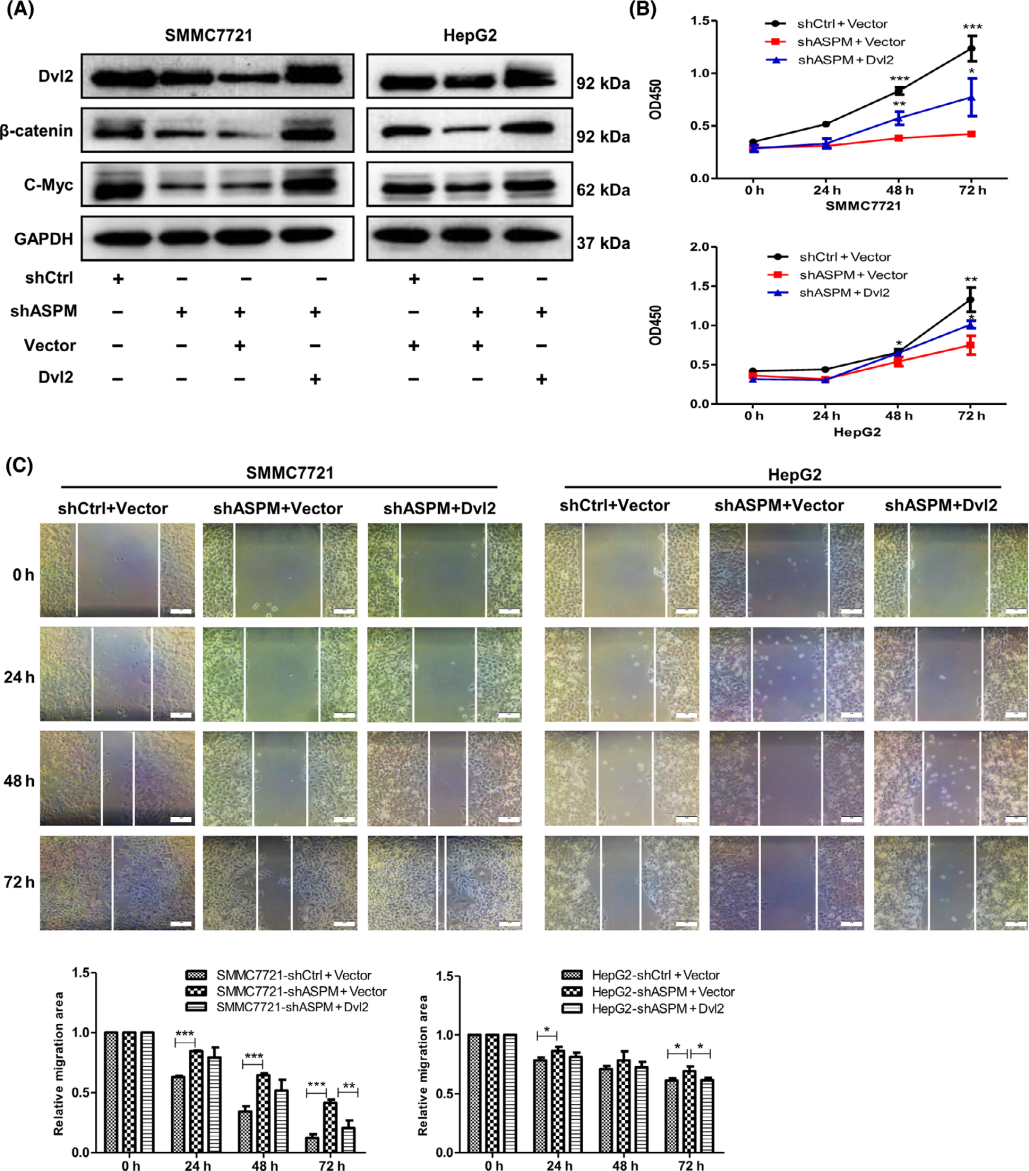
Overexpression of Dvl2 rescues the phenotypes of ASPM‐KD HCC cells. (A) Control or shASPM SMMC7721 and HepG2 cells were transfected with empty vector or Dvl‐2 expression vector, after which β‐catenin, Dvl‐2 and c‐Myc were detected by western blot. (B) Proliferation curve of control or shASPM SMMC7721 and HepG2 cells after transient transfections with empty vector or Dvl2 expression vector. Data are shown as means ± SD (*n* = 3 in each assay, Student's *t*‐test). *P ≤ 0.05, **P ≤ 0.01, ***P ≤ 0.001 vs. shASPM + Vector. (C) Representative images of scratch wound assays of control or shASPM SMMC7721 and HepG2 cells after transient transfections with empty vector or Dvl‐2 expression vector. Scale bars: 100 μm. Data are shown as means ± SD (*n* = 3 in each assay, Student's *t*‐test). *P ≤ 0.05, **P ≤ 0.01, ***P ≤ 0.001 vs. shASPM + Vector.

Next, we ectopically expressed Dvl‐2 in ASPM‐silenced SMMC‐7721 and HepG2 cells. Indeed, overexpression of Dvl2 in shASPM HCC cells rescued the protein level of Dvl2, as well as β‐catenin and c‐Myc (Fig. 6A). Correspondingly, the inhibitory effect of ASPM deletion on cell proliferation and migration was significantly restored by ectopic expression of Dvl2 (Fig. 6B,C). Collectively, these results indicate that ASPM binds with Dvl2 and stabilizes the protein level of Dvl2, thereby enhancing Wnt pathway activity.

### ASPM stabilizes Dvl2 by antagonizing the autophagy system

**Fig. 7:**
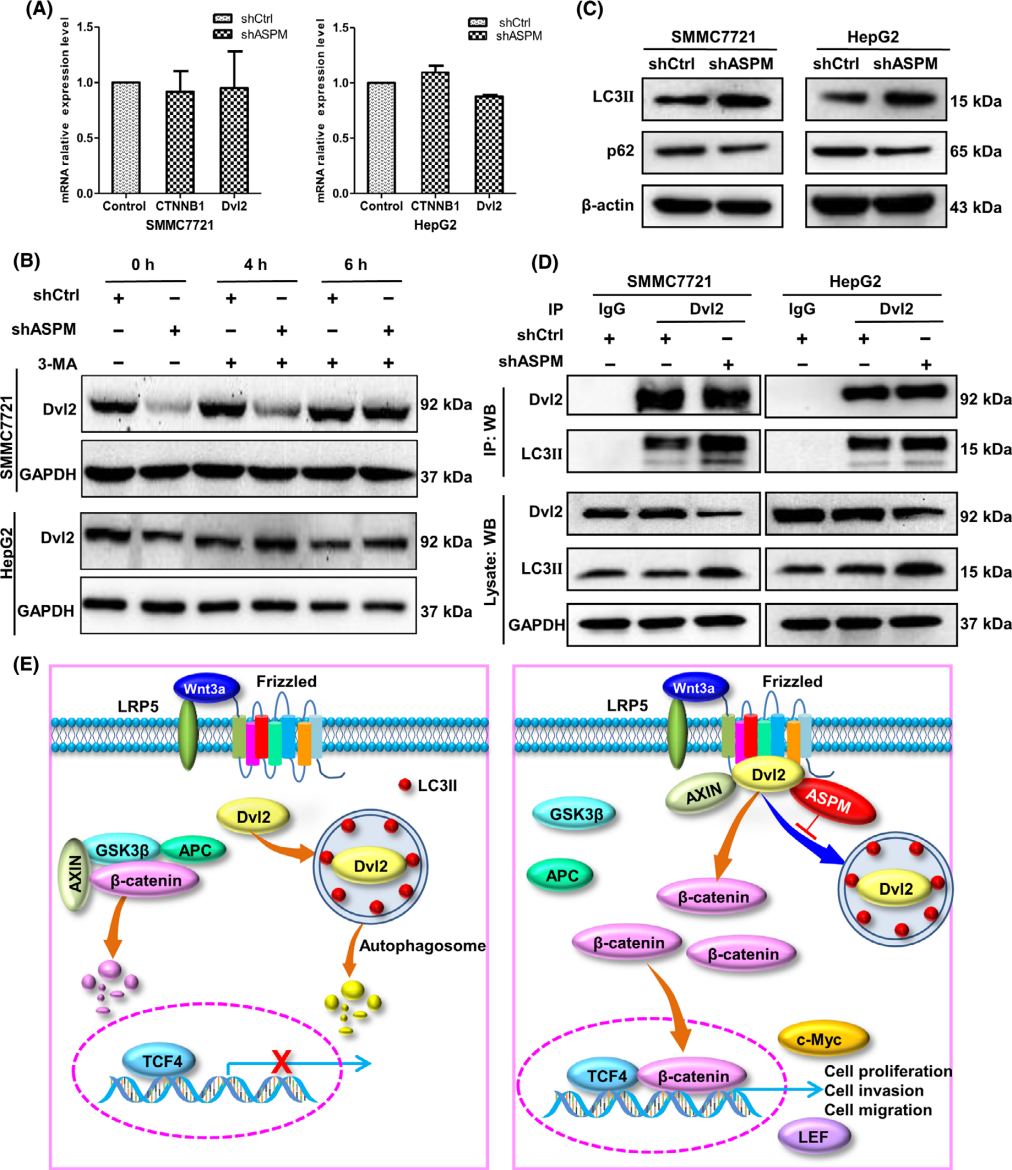
ASPM stabilizes Dvl2 via antagonizing the autophagy system. (A) The transcript levels of *Dvl2* and *CTNNB1* in control or shASPM SMMC7721 and HepG2 cells measured by qRT‐PCR. Data are means ± SD (*n* = 3 in each assay, Student's *t*‐test). (B) Western blot analysis of Dvl2 protein levels in control or shASPM SMMC7721 and HepG2 cells at 0, 4 and 6 h after treatment with autophagy inhibitor 3‐MA (10 mM), respectively. (C) Western blot analysis of LC3II and p62 protein levels in control or shASPM SMMC7721 and HepG2 cells. (D) Co‐IP analysis between Dvl2 and LC3II in control or shASPM SMMC7721 and HepG2 cells. (E) The proposed model illustrates how ASPM promotes HCC progression. Left panel: without ASPM, Dvl2 is degraded by the autophagic lysosomal pathway, and β‐catenin is destroyed by destruction complex, which prevents the accumulation of β‐catenin protein in the cytoplasm and thereby the inactivation of the Wnt/β‐catenin pathway. Right panel: when ASPM binds with Dvl2, it stabilizes Dvl2 protein via antagonizing the autophagy system, thereby resulting in the translocation of β‐catenin into the nucleus and augmenting Wnt/β‐catenin signaling.

ASPM silencing in HCC cells resulted in the decrease of Dvl2 protein level, but mRNA expression of *Dvl2* and *CTNNB1* (encoding β‐catenin) was unchanged (Fig. 7A), indicating that the deletion of ASPM might increase Dvl2 protein degradation. Autophagic lysosomal pathway is responsible for the degradation of cellular protein, and a recent study revealed that autophagy regulated Dvl2 degradation in skeletal muscle regeneration [28]. We thus considered the possibility that ASPM may function in HCC cells in a similar way. We then treated HCC cells with autophagy inhibitor 3‐methyladenine (3‐MA) and found that the protein levels of Dvl2 were significantly restored at 6 h in ASPM‐silenced SMMC‐7721 cells and at 4 h in ASPM‐silenced HepG2 cells (Fig. 7B), suggesting that Dvl2 decrease in ASPM‐deficient HCC cells may involve an autophagy‐dependent pathway. Importantly, we found that the autophagy markers LC3II and p62 were significantly increased and decreased, respectively, in shASPM SMMC7721 and HepG2 cells (Fig. 7C). Co‐IP assay demonstrated that Dvl2 interacted with LC3II, and the deletion of ASPM increased this interaction (Fig. 7D). Taken together, these results reveal that ASPM KD increases the autophagy‐mediated Dvl2 degradation, so ASPM can stabilize Dvl2 protein by binding with Dvl2 and preventing the autophagy‐mediated degradation of Dvl2. The proposed model that illustrates how ASPM promotes HCC progression is shown in Fig. 7E.

### Correlation between ASPM and key members of the Wnt/β‐catenin pathway in liver cancer clinical samples

To validate the correlation between ASPM and the Wnt/β‐catenin pathway, we also examined the mRNA expression levels of Dvl‐2, CTNNB1, TCF4 and LEF1 in liver tumor tissues. Correlation studies revealed that the expression of ASPM was positively associated with the levels of Dvl‐2, CTNNB1, TCF4 and LEF1, respectively (all *P* < 0.01; Table S4). Altogether, the results suggest that ASPM exerts its tumor‐promoting function through activating the Wnt/Dvl2/β‐catenin pathway in liver cancer.

## Discussion

In this work, we found that ASPM is highly expressed, at both mRNA and protein levels, in liver cancer tissues, especially in early recurrent HCC, and up‐regulation of ASPM is significantly related to malignant clinicopathological features and short overall survival in patients with liver cancer. Moreover, we demonstrated that ASPM plays important roles in HCC proliferation, invasion, migration and EMT by activating the Wnt/β‐catenin pathway via preventing the autophagy‐mediated degradation of Dvl2. Therefore, we conclude that ASPM is a novel oncogene in liver cancer and could serve as a potentially valuable therapeutic target for liver cancer.

ASPM is a large mitotic spindle protein of 410 kDa, and its functions in mammalian cells is not yet fully elucidated [29]. The original research showed that ASPM was specifically expressed in the embryonic cerebral cortex of mouse and regulated the symmetric mitosis of neural progenitor cells of the mammalian brain [7, 30]. Subsequent study found that ASPM mRNA was broadly expressed in the embryonic and adult tissues, and the expression level was much higher in fetal tissues than in adult tissues, implying that ASPM may play a role in cell proliferation [11]. Recently, aberrant expression of ASPM has been reported in various types of human cancers, and ASPM has been identified as a potential marker for many tumors [13, 15, 16, 20]. In this study, we revealed that ASPM was highly expressed not only in different histological types of liver cancer but also in different phases of HCC (primary HCC and recurrent HCC), as well as in HCC‐derived cell lines. Therefore, we speculated that ASPM may act as a pan‐cancer driver gene and present a promising broad‐spectrum anticancer molecular target.

We also found that up‐regulation of ASPM in HCC was associated with vascular invasion and tumor embolus, indicating that ASPM may be involved in HCC invasion and metastasis. Although previous study mentioned the potential role of ASPM in tumor metastasis [13], the underlying mechanism is unknown. Our functional experiments demonstrated that ASPM could significantly enhance the invasive and migratory capacities of HCC cells. EMT is the initial and necessary step of the metastatic cascade of many cancers [31], which is controlled by many EMT‐related transcriptional factors, such as Slug, Twist and Snail. Through EMT marker detection and EMT‐related transcriptional factor analysis, we verified that ASPM promotes HCC invasion and migration via inducing EMT of HCC cells. Therefore, our data are the first to illustrate that ASPM could induce EMT in HCC.

The Wnt/β‐catenin signaling pathway plays essential roles from embryo development to tissue homeostasis, and its deregulation is involved in tumorigenesis and many other diseases [32, 33]. The hallmark of the canonical Wnt signaling activation is the accumulation of β‐catenin in the cytoplasm and then translocation into the nucleus, where β‐catenin forms a complex with TCF4/LEF1 and regulates the transcription of multiple genes, such as *c‐Myc*, *Axin2* and *LEF1*. Dvl2, a masterful conductor of the Wnt pathway, inhibits destruction complex‐induced degradation of β‐catenin and thereby leads to β‐catenin accumulation and Wnt signal activation [26, 27]. The Wnt/β‐catenin signaling is the most frequently deregulated pathway in HCC [24], and aberrant activation of Wnt signaling contributes to HCC initiation, progression, invasion, metastasis and recurrence, resulting in low survival rates [34]. Around 40–70% of HCCs show elevated Wnt/β‐catenin signaling activity, and *CTNNB1* (encoding for β‐catenin) mutation frequencies increase significantly with HCC progression [35].

Recent work has identified ASPM as a positive regulator of Wnt signaling pathway [13, 25]. In the developing brain, ASPM facilitated Wnt‐mediated neurogenesis programs [25]. In this study, we found that ASPM KD caused a significant decrease of Dvl2 and β‐catenin protein abundance. Moreover, we also revealed that the deletion of ASPM led to the protein level reduction of Wnt downstream components, TCF4, LEF1 and c‐Myc. Both *c‐Myc* and *LEF1* are Wnt target genes. It is well‐known that c‐Myc contributes to cell malignant transformation and tumorigenesis by promoting protein synthesis, uncontrolled cell proliferation and dysregulated tumor cell metabolism [36]. LEF1 is a transcription factor that cooperates with β‐catenin to enable Wnt target gene transcription. In this study, we speculated that ASPM activates Wnt/β‐catenin to cause LEF1 level increase; meanwhile, enhanced LEF1 augments Wnt target gene transcription, such as c‐Myc and LEF1. Thus, ASPM–Dvl2–LEF1–c‐Myc may form positive feedback to promote HCC progression. In addition, the Wnt signaling pathway also regulates the expression of transcription factors essential to EMT, such as Slug, Twist and Snail [37]. Therefore, EMT of HCC cells induced by ASPM may be attributed to Wnt pathway activation. Taken together, these data illustrate that ASPM acts as a novel oncoprotein in HCC by activating the Wnt/β‐catenin pathway, thereby promoting HCC EMT and progression.

We observed that ASPM interacted with and stabilized Dvl2 to activate the Wnt/β‐catenin pathway. Dvl2 is the hub of Wnt signaling, which relays Wnt signals from Wnt receptors to β‐catenin. The decrease of Dvl2 protein abundance abrogates canonical Wnt signaling, and the stability of Dvl proteins is closely regulated [38]. Autophagic lysosomal pathway is responsible for the degradation of cellular protein, and autophagy regulates canonical Wnt/β‐catenin signaling through regulation of Dvl2 levels [28, 39]. LC3 and p62 are the most frequently used markers of autophagosomes, and LC3II increases represent the induction of autophagy, while increased p62 levels are associated with autophagy inhibition [40].

In this study, we found that deletion of ASPM in HCC cells caused a significant LC3II increase and p62 decrease, respectively, which suggests that autophagy is induced in ASPM‐deficient HCC cells. Meanwhile, Dvl2 interacted with LC3II, and KD of ASPM significantly promoted this interaction, indicating that LC3II targeted Dvl2 and therefore accelerated the autophagy‐mediated degradation of Dvl2 in ASPM‐deficient HCC cells. A recent report verified that GFP‐LC3^+^/Dvl2 puncta formed when the Dvl2 plasmid was transfected into C2C12 cells [27]. Furthermore, the Dvl2 rescue detected in shASPM HCC cells treated with the autophagy inhibitor 3‐MA confirms that ASPM is necessary to protect Dvl2 from autophagy‐mediated degradation. These results together suggest that ASPM interacts with Dvl2 and thereby prevents autophagy‐mediated Dvl2 degradation and increases its stability.

ASPM is a large protein, and its regulation is complex. ASPM expression was regulated via the PKR‐p38 MAPK signaling pathway in HCV NS5A‐expressing mouse hepatocytes [41]. However, recent studies revealed that transcription factor FoxM1 bound to the promoter of ASPM and activated the transcription of ASPM directly in glioma cells and gastric cancer cells [42, 43]. The regulation of ASPM expression in liver or in HCC cells remains unclear, and future study is required to investigate the underlying mechanism.

In summary, this study revealed that abnormal expression of ASPM plays a pivotal role in the progression of HCC through interacting with Dvl2, physically preventing the formation of Dvl2 and LC3II protein complex, and augmenting the Wnt–ASPM–Dvl‐2–β‐catenin signaling axis, which not only sheds a new light on HCC progression but also provides a potential target for HCC treatment.

## Conflict of interest

The authors declare no conflict of interest.

## Author contributions

HZ and JZ conceived and designed the study. HZ, ZL and CZ collected the liver samples. XY, LZ, ZL, PZ, PW and JF performed the experiments and acquired the data. YM, HY, YX and GJ collected and analyzed the data. HZ, XY and LZ wrote the paper.

## Supporting information

**Table S1**. Donor characteristics of human liver samples.
**Table S2**. Primers for quantitative real‐time polymerase chain reaction.
**Table S3**. ASPM mRNA expression levels of normal and tumor tissues in different histological types or different phases of liver cancer patients
**Table S4**. Correlation between the mRNA expression levels of ASPM and Wnt/β‐catenin pathway key members in liver tumor tissues.

## Data Availability

The data that support the findings of this study are available from the corresponding author upon reasonable request.
