# Supplementary material for: ASPM promotes hepatocellular carcinoma progression by activating Wnt/β‐catenin signaling through antagonizing autophagy‐mediated Dvl2 degradation
Source: FEBS Open Bio. 2021 Sep 14;11(10):2784–99. doi: 10.1002/2211-5463.13278 (PMC8487047; doi:10.1002/2211-5463.13278)
Supplement: Supplementary file 1 — Table S1. Donor characteristics of human liver samples. Table S2. Primers for quantitative real‐time polymerase chain reaction. Table S3. ASPM mRNA expression levels of normal and tumor tissues in different histological types or different phases of liver cancer patients Table S4. Correlation between the mRNA expression levels of ASPM and Wnt/β‐catenin pathway key members in liver tumor tissues. [file FEB4-11-2784-s001.docx]

**Supplementary Tables S1-S4**

**Supplementary Table S1** **Donor characteristics of human liver samples**

| Variables | Group | Number | Percentage (%) |
| --- | --- | --- | --- |
| Gender | Male | 79 | 77.5 |
|  | Female | 23 | 22.5 |
| Age (years) | 20-45 | 24 | 23.5 |
|  | 46-60 | 49 | 48.0 |
|  | 61-75 | 29 | 28.4 |
| Smoking | Non-smoking | 66 | 64.7 |
|  | Smoking | 36 | 35.3 |
| Drinking | Non-drinking | 64 | 62.7 |
|  | Drinking | 38 | 37.3 |
| Medical Diagnosis | HBV-HCC | 74 | 72.5 |
|  | HBV-RHCC | 10 | 9.8 |
|  | HCC | 4 | 3.9 |
|  | ICC | 7 | 6.9 |
|  | MLC | 7 | 6.9 |

HBV-HCC: HBV-related primary hepatocellular carcinoma; HBV-RHCC: HBV-related recurrent hepatocellular carcinoma; HCC: primary hepatocellular carcinoma; ICC: intrahepatic cholangiocarcinoma; MLC: metastatic liver cancer.

**Supplementary Table S2** **Primers for quantitative real-time polymerase chain reaction**

| Gene | Forward primer (5’→3’) | Reverse (5’→3’) | Fragment Size（bp） |
| --- | --- | --- | --- |
| *GAPDH* | AACAGGGTGGTGGACCTCAT | GGAGGGGAGATTCAGTGTGG | 153 |
| *ASPM* | TGCAGTGGGTGAACATGAAAA | CGAAGAGGGTGTTACCTCGTTT | 130 |
| *Dvl-2* | TCAGCAGCGTCACAGATTCC | GTCTCCCCGCTCATTGCTC | 116 |
| *CTNNB1* | CATCTACACAGTTTGATGCTGCT | GCAGTTTTGTCAGTTCAGGGA | 150 |
| *TCF4* | CAAGCACTGCCGACTACAATA | CCAGGCTGATTCATCCCACTG | 145 |
| *LEF1* | TGCCAAATATGAATAACGACCCA | GAGAAAAGTGCTCGTCACTGT | 150 |

**Supplementary Table S3** **ASPM mRNA expression levels of normal and tumor tissues in different histological types or different phases of liver cancer patients**

| Medical Diagnosis Group | Number | | Median | | Range（P_25_-P_75_） | |
| --- | --- | --- | --- | --- | --- | --- |
|  | Normal | Tumor | Normal | Tumor | Normal | Tumor |
| HBV- HCC | 71 | 74 | 0.001 | 0.005 | 0-0.002 | 0.001-0.010 |
| HBV-RHCC | 9 | 10 | 0.001 | 0.005 | 0-0.002 | 0.002-0.030 |
| HCC | 4 | 4 | 0.001 | 0.007 | 0-0.006 | 0-0.016 |
| ICC | 6 | 3 | 0.000 | 0.013 | 0-0.001 | —— |
| MCC | 6 | 5 | 0.002 | 0.004 | 0-0.006 | 0.001-0.017 |

HBV-HCC: HBV-related primary hepatocellular carcinoma; HBV-RHCC: HBV-related recurrent hepatocellular carcinoma; HCC: primary hepatocellular carcinoma; ICC: intrahepatic cholangiocarcinoma; MLC: metastatic liver cancer.

**Supplementary Table S4 Correlation between the mRNA expression levels of ASPM and Wnt/β-catenin pathway key members in liver tumor tissues.**

| Tumor | | ASPM | Dvl-2 | CTNNB1 | TCF4 | LEF1 |
| --- | --- | --- | --- | --- | --- | --- |
| ASPM | *r* | 1.000 | .355^**^ | .519^**^ | .495^**^ | .454^**^ |
|  | n | 96 | 95 | 95 | 94 | 96 |
| Dvl-2 | *r* |  | 1.000 | .390^**^ | .458^**^ | .389^**^ |
|  | n |  | 95 | 95 | 93 | 95 |
| CTNNB1 | *r* |  |  | 1.000 | .723^**^ | .551^**^ |
|  | n |  |  | 95 | 93 | 95 |
| TCF4 | *r* |  |  |  | 1.000 | .672^**^ |
|  | n |  |  |  | 94 | 94 |
| LEF1 | *r* |  |  |  |  | 1.000 |
|  | n |  |  |  |  | 96 |

**P*<0.05*,* ***P*<0.01.
